# The AKT inhibitor AZD5363 is selectively active in PI3KCA mutant gastric cancer, and sensitizes a patient-derived gastric cancer xenograft model with PTEN loss to Taxotere

**DOI:** 10.1186/1479-5876-11-241

**Published:** 2013-10-02

**Authors:** Jing Li, Barry R Davies, Sufang Han, Minhua Zhou, Yu Bai, Jingchuan Zhang, Yan Xu, Lily Tang, Huiying Wang, Yuan Jie Liu, Xiaolu Yin, Qunsheng Ji, De-Hua Yu

**Affiliations:** 1 Innovation Center China, AstraZeneca, No. 199 Liangjing Road, Zhangjiang Hi-Tech Park, Shanghai 201203, China; 2 Oncology iMED, AstraZeneca, Alderley Park, Macclesfield SK0 4TG, UK

## Abstract

**Introduction:**

Activation of the PI3K/AKT pathway is a common phenomenon in cancer due to multiple mechanisms, including mutation of PI3KCA, loss or mutation of PTEN, or over-expression of receptor tyrosine kinases. We recently developed a novel AKT kinase inhibitor, AZD5363, and demonstrated that HGC27, a cell line harboring both PI3KCA mutation and PTEN loss, displayed the greatest sensitivity to this AKT inhibitor *in vitro* and *in vivo*.

**Case preparation:**

To further elucidate the correlation between AZD5363 response and genetic alterations in gastric cancer (GC) and identify GC patients with both PI3KCA mutations and PTEN loss, we investigated the effects of pharmacological inhibition of AKT on a panel of 20 GC cell lines and genetic aberrations in tumor samples from a cohort of Chinese GC patients. We demonstrated that GC cells with PI3KCA mutations were selectively sensitive to AZD5363. Disease linkage studies showed that PI3KCA activating mutations or PTEN loss were found in 2.7% (4/150) and 23% (14/61) of Chinese GC patients respectively. To further dissect the role of PI3KCA mutation and PTEN loss in response to AKT inhibition, we tested the antitumor activity of AZD5363 in two patient-derived GC xenograft (PDGCX) models harboring either PI3KCA mutation or PTEN loss. Our data indicated that AZD5363 monotherapy treatment led to a moderate response in the PI3KCA mutant PDGCX model. Whilst monotherapy AZD5363 or Taxotere were ineffective in the PTEN negative PDGCX model, significant anti-tumor activity was observed when AZD5363 was combined with Taxotere.

**Conclusion:**

Our results indicated that PI3KCA mutation is an important determinant of response to AKT inhibition in GC and combination with AZD5363 can overcome innate resistance to Taxotere in a PTEN loss PDGCX model. It is suggested that AKT inhibitor is an attractive option for treatment of a new segment of GC patients with aberrant PI3K/AKT signaling.

## Introduction

Gastric cancer (GC) is one of the most lethal malignancies and the second leading cause of cancer death [1]. The estimated global incidence and mortality of GC in 2011 were 990,000 and 737,000 cases respectively, accounting for approximately 8% of total cancer cases and 10% of annual cancer deaths worldwide [2]. Geographically, GC is far more prevalent in developing countries compared to developed nations. Nations of high prevalence include Eastern Asia, Central and Eastern Europe, and South America, accounting for ~70% of the total cases. The conventional treatments for GC include surgery, radiotherapy, and chemotherapy [3]. Although these modalities are able to prolong the overall survival of patients with early disease by 20-35%, they have very limited efficacy in treating patients with advanced GC, conferring a median survival time in the range of 6–11 months, with considerable treatment-related toxicities [4]. Due to the complexity of the molecular signaling pathways involved in carcinogenesis and the lower prevalence in western countries, the development of targeted therapies for GC has lagged compared to many other cancer indications. Overexpression/amplification of Her2 has been observed in 10-38% GC patients [5]. The recent phase III ToGA trial involving 3,800 GC patients indicated that the combination of trastuzumab and chemotherapy in Her2+ GC patients led to a significantly higher overall response rate (ORR), 47% versus 35%, significantly longer progression free survival (PFS) interval, 6.7 months versus 5.5 months, and significantly longer OS duration, 13.8 months versus 11.1 months compared to the chemotherapy arms respectively [6]. This positive result led to the approval of trastuzumab as the first molecularly targeted therapeutic agent for GC in both the U.S. and Europe.

AKT is a serine/threonine protein kinase that plays a central role in the signaling network involving PI3K and mTOR, and which regulates multiple cellular processes including glucose metabolism, apoptosis, cell proliferation, transcription and cell migration. Under normal conditions, this signaling network can be activated by many receptors, including members of the epidermal growth factor receptor (EGFR) and vascular endothelial growth factor receptor (VEGFR) families and their ligands. The activation of the PI3K/AKT/mTOR signaling network has been commonly observed in many human cancers, and can be triggered by a variety of mechanisms including overexpression of upstream receptors, activating PI3KCA mutations, loss of PTEN function, and overexpression or activation of AKT [5]. For instance, the increased phosphorylations of AKT and mTOR have been observed in 80% of [7] and 47%–64% of GC patients [7-10]. Further investigations have demonstrated that the activation of the AKT/PI3K network can be attributed to overexpression of upstream receptors (20%–30%), PI3KCA activating mutations (4%–36%) [11,12] and PTEN loss (20%–36%) [11,13]. A recent study by Linos et al indicated that PTEN was lost in the majority of Her2 positive GC cases [14]. These observations provide a possible explanation for the observed clinical resistance of Her2 positive breast cancer patients to current anti-Her2 therapies, including Trastuzumab and lapatinib. This also suggests a rationale for the design of new combination therapies through dual targeting of the Her2 and PI3K–Akt–mTOR networks. Besides the involvement in resistance to anti-Her2 therapies, the importance of the PI3K–Akt–mTOR network in the resistance to chemotherapies in GC has been documented by a number of studies [12,15]. In one such study, reduction of basal AKT activity by ectopic expression of PTEN sensitized GC cells to anti-cancer chemotherapy agents. When primary tumor tissues from GC were tested for their chemotherapeutic sensitivity *in vitro*, the association between activated AKT and increased resistance to multiple chemotherapeutic agents including 5-fluorouracil, doxorubicin, mitomycin C, and cisplatin was found [16].

We previously reported the development of a novel AKT kinase inhibitor AZD5363, and found that cells with both PI3KCA mutation and PTEN loss were highly sensitive to treatment using AZD5363 [17]. In this study, we further investigated the correlation between the sensitivity of a panel of gastric cell lines to AZD5363 *in vitro* and their genetic aberrations. Using PDGCX models derived from patient GC tissues, we further confirmed a role for PI3KCA activating mutations and PTEN loss in sensitizing tumors to AKT inhibition.

## Materials and methods

### Cell culture reagents, and proliferation assay

Human GC cell lines PAMC82 cells were obtained from Beijing tumor hospital (Beijing, China). GTL-16, 23132/87 cells were provided by AztraZeneca tissue culture unit (Alderley Park, UK). NCI-N87, SNU-1, SNU-5, SNU-16, HS746T and AGC were purchased from American type culture collection (ATCC, American). KATOIII and HGC27 were obtained from Europe collection of Cell Cultures (ECACC, UK). NUGC-4, IM95 m, MKN-1, OCUM-1, MKN-74, AZ-521 cells were obtained from Japanese Collection of Research Bioresources Cell Bank (JCRB, Japanese). NUGC-3 cells were obtained from Beijing University (Beijing, China). SNU-261, SNU-484, SNU-601, SNU-620, SNU-638 and SNU-668 cells were obtained from Korean cell line bank. IM95 m and HS746T cells were cultured in DMEM medium with 10% FBS and 10 ug/ml insulin. OUCM-1 cells were cultured in DMEM medium containing 10% FBS and 1% Na Pyruvate. All other cells were maintained in RPMI 1640 (Invitrogen) supplemented with 10% FBS and 2 mM L-Glutamine. All cells were maintained in a humidified incubator with 5% CO_2_ at 37°C. The structure and synthesis of AKT inhibitor AZD5363 [(S)-4-amino-N-[1-(4-chlorophenyl)-3-hydroxypropyl]-1-(7H-pyrrolo [2, 3-d]pyrimidin 4-yl)piperidine-4-carboxamide has been described previously [17].

Cell growth rate was measured by a MTS assay. Briefly, cells seeded at 1000-2000/well density in 96 well plates were cultured overnight, and then treated with AZD5363 at different concentrations for 72 hrs. CellTiter 96® Aqueous One Solution Reagent (Promega, Madison, WI) was added to each well according to the manufacturer’s instructions. After 2 hours in culture the cell viability was determined by measuring the absorbance at 490 nm using Safire 2 plate-reader (Tecan, Switzerland).

### Patients and tumor samples

The present study included 116 patients with GC who underwent surgery between 2007 to 2011 at the Renji Hospital, Shanghai, China. All patients underwent radical surgical resection, followed by standard chemotherapy for the majority of the patients. Histologic subtype according to Lauren’s classification was determined after a review of tumor sections by two trained pathologists. This study was approved by the institutional review board at Renji Hospital.

### Tissue microarray construction

GC tissue samples were fixed in buffered 4% formalin for a minimum of 24 hours and embedded in paraffin. The construction of tissue microarray (TMA) follows standard procedures as previously described [18].

### Immunohistochemistry (IHC)

The slides were baked at 56°C for 1 hour, then de-paraffinized in xylene and hydrated through graded series of alcohols. Antigen retrieval was done in pressure cooker for 5 min using Citrate pH6, Target Retrieval Solution (Dako, Glostrup, Denmark). After cooling to room temperature, endogenous peroxidase activity was blocked by Peroxidase Blocking Reagent (Dako) for 5 minutes. The sections were then incubated with rabbit monoclonal antibody against PTEN (Cell Signaling Technology, USA) for 1 hour at room temperature. Then the secondary anti-rabbit antibody (Dako, Envision) was applied to the sections for 30 minutes at room temperature. After rinsed with TBST, the slides were treated with DAB substrate-chromagen (Dako), counterstained with haematoxylin, dehydrated and mounted with coverslips. Scoring was established as follows: 0, if absence of staining was observed; 1+, if the tumor cells had weak staining; 2+, if tumor cells had moderate staining; and 3+ if tumor cells had strong staining. Tumors with 1+, 2+, and 3+ expression were interpreted as positive and tumors with no expression (0 score) were interpreted as negative. Given the heterogeneity of protein expression in tumor cells, the highest scoring from either one of TMA cores was counted as the final result. To minimize impact of intratumoral heterogeneity, case-matched whole sections of negatively-scored patient TMA samples were re-evaluated by IHC. All slides were independently evaluated by two pathologists (X. Y. and L. Z.) who are blind to patients’ clinical data. The two pathologists discussed and reached final consensus result for each case.

### Western blot analysis

Frozen tumor fragments were homogenized in liquid nitrogen using a mortar and pestle and then lysed in RIPA buffer (Pierce, #89901) containing Halt protease & phosphatase inhibitor cocktail (Pierce,78447). Soluble proteins were quantified by BCA protein level detection kit (Pierce,#23227 ), then soluble proteins (80 μg) subjected to SDS-PAGE followed by immunoblotting. Antibody incubation was conducted overnight at 4°C. Antibodies were obtained from the following sources: phosphor-Akt (S473) (Dako, cat.M3628), phosphor-PRAS40(Tyr436) (Cell Signaling Technologies, cat.2997), Phospho-S6 Ribosomal protein (Ser235/236) (Cell Signaling Technologies, cat.2211), AKT (Cell Signaling Technologies, cat.9272), PRAS40(Cell Signaling Technologies, cat.2610), S6 Ribosomal Protein (54 D2) (Cell Signaling Technologies, cat 2317), and GAPDH (Cell Signaling Technologies, cat.2118). Secondary antibodies were applied and immunoreactive proteins were visualized using “;SuperSignal West Dura”; Extended Duration Substrate according to the manufacturer’s instructions (Thermo, #34076).

### Sanger sequencing

PCR was performed in a 25 μL reaction mix containing 1× AmpliTaq Gold® 360 Master Mix (Life Technologies), 200 μM of each primer, and 5 μL of genomic DNA. PI3K, Braf and Kras genes were amplified using the following primers: PI3KCA exon 10 forward 5′-GTCTTAGATTGGTTCTTTCCTG-3′, PI3KCA exon 10 reverse 5′-GCATTTAATGTGCCAACTACC-3′; PI3KCA exon 21 forward 5′- TTTGTCTACGAAAGCCTCTCTA-3′, PI3KCA exon 21 reverse 5′- CCATCACTTTTTCCTTCTCCAT-3′. Braf exon 15 forward 5′- TTCATAATGCTTGCTCTGATAGGAAAATG-3′, Braf exon 15 reverse 5′- GAACACTGATTTTTGTGAATACTGGGAACT-3′, and Kras exon 2 forward 5′- GTACTGGTGGAGTATTTGATAGTG-3′, Kras exon 2 reverse 5′-GAGAGTGAACATCATGGACCCTG-3′. The PCR cycling conditions were: 10-min incubation at 95°C, followed by 40 cycles of 94°C for 30 s, 60°C for 30 s, 72°C for 60 s, and then a final incubation at 72°C for 10 min. The resulting PCR products were digested with ExoSAP-IT reagent (Affymetrix, Cleveland, Ohio, USA), and then sequenced in forward and reverse directions with BigDye Terminator Kit (Life Technologies) and an ABI 3730XL DNA analyzer (Life Technologies) following the manufacturer’s instructions. The sequencing data were analyzed for mutations after assembly and quality calling with SeqScape sequence analysis software (version 2.5; Life Technologies).

### Allele specific polymerase chain reaction (ASPCR)

Human PI3K Gene Mutation Fluorescence Polymerase Chain Reaction (PCR) diagnostic kit (Amoy Diagnostics, Xiamen, China) was used for the Pi3KCA mutation detection in this study. This kit detects the 5 most common Pi3KCA mutations: E542K, E545D, E545K, H1047R, H1047L. All experiments were performed following the manufacturer’s instructions. Briefly, 5 μL DNA was added to 45 μL of the PCR master mix for each assay, which contained PCR primers, fluorescent probes, PCR buffer, and DNA polymerase. The PCR cycling conditions were: 5-min incubation at 95°C, followed by 15 cycles of 95°C for 25 s, 64°C for 20 s, 72°C for 20 s, and then 26 cycles of 93°C for 25 s, 60°C for 35 s, 72°C for 20 s. Fluorescent signal was collected from FAM and HEX channels. Genotypes were determined according to threshold cycles (Ct) as indicated in the manufacturer’s instructions.

### Array comparative genomic hybridization (aCGH)

DNA was extracted using the QIAamp DNA Mini Kit (Qiagen) according to the manufacturer’s instructions. Array comparative genomic hybridization (aCGH) analysis was performed using the Agilent Human Genome 244 K Microarray Chip (Agilent Technologies) with pooled human genomic DNA (Promega) as a reference. Labeling, hybridization, washes, and data analysis were performed according to the protocol provided by Agilent (Protocol version 4.0, June 2006). Graphical overviews were obtained using the CGH Analytics software (version 3.5, Agilent Technologies).

### Multiplex ligation-dependent probe amplification (MLPA)

MLPA assay was carried out according to the manufacturer’s instructions with the PTEN kit P225-A1 (MRC-Holland, Netherlands). Fragment analysis was performed with 1 μl MLPA PCR products by caplillary electrophoresis on an ABI3730XL DNA analyzer and analyzed using Genotyper software v4.0 (Life technologies, USA). Each exon copy number of PTEN was determined by calculating ratio of normalized test and control peak heights in each run.

### *In vivo* efficacy studies using patient-derived GC xenograft (PDGCX) mouse models

Eight- to 10-week-old female nude (nu/nu) mice (Vital River, Beijing) were used for development of PDGCX mouse models and *in vivo* efficacy studies. All experiments using immunodeficient mice were carried out in accordance with the guidelines approved by Institutional Animal Care and Use Committees (IACUC). PDGCX mouse models were established by directly implanting fresh surgical tumor tissue into immunodeficient mice. Prior written informed consents were obtained from all patients who contribute his/her tumor tissues and the study protocol was approved by the local hospital ethics committee. After establishment and characterization of PDGCX models, PDGCX tissues were harvested and frozen in medium with 90% serum and 10% DMSO in liquid nitrogen. For the anti-tumor efficacy studies, PDGCX tissue fragments (~15 mm^3^) were implanted subcutaneously via Trocar needle into female nude mice subcutaneously. Tumor-bearing mice with a tumor size range of 100 to 200 mm^3^ were randomly divided into control and treatment groups (8 animals per group) for treatment. AZD5363 was solubilized in 10% DMSO 25% w/v Kleptose HPB (Roquette) buffer and dosed by oral gavage. Taxotere (Sanofi-Aventis) solubilized in 2.6% ethanol was administrated by intravenous injection once weekly. For the combination treatment, Taxotere was injected one hour in the PDGCX model before the oral dose of AZD5363. The control group received the DMSO/Kleptose buffer alone, twice daily by oral gavage. Subcutaneous tumors in nude mice and mouse body weight were measured twice weekly. Tumor volumes were calculated by measuring 2 perpendicular diameters with calipers [formula: V = (length × width^2^)/2]. Percentage tumor growth inhibition [%TGI = 1-(change of tumor volume in treatment group/change of tumor volume in control group) × 100] was used for the evaluation of antitumor efficacy. Statistical significance was evaluated using a one-tailed, two-sample t test. P < 0.05 was considered statistically significant.

## Results

### GC cells harboring PI3KCA mutations were selectively sensitive to the AKT inhibitor AZD5363

To explore the potential application of AZD5363 in GC, a panel of 22 GC lines was tested for anti-proliferative sensitivity using an *in vitro* cell growth assay. Cells seeded in 96 well plates were cultured overnight, and then treated with AZD5363 at different concentrations for 72 hrs. Cell growth rate was measured by a MTS assay. As shown in Table 1, 6 out of the 22 tested cell lines displayed sensitivity to AZD5363 with IC50’s lower than 3 μM [17]. Further analysis of the known genetic lesions in the GC cell panel indicated that 4 of the sensitive cell lines harbored the PI3KCA E542K mutation, one of the major PI3KCA activating mutations reported previously [19]. Interestingly, HGC27, the most sensitive line amongst the 4 PI3KCA mutant cell lines, contained not only PI3KCA mutation, but also PTEN loss. As we reported recently [17], when nude mice bearing HGC27 xenograft tumors were treated with AZD5363, complete tumor regression was achieved. Together, these data suggest that activation of the PI3K/AKT pathway by PI3KCA mutations or PTEN loss drives tumorigenesis in GC, potentially defining a new molecular segment for AKT-based targeted therapies.

**Table 1:** AZD5363 inhibits proliferation of a subset of GC cell lines in vitro

**Cell line**	**Genetic background**		**GI50 (μmol/L)**
	**PIK3CA**	**PTEN**	
HGC27	E542K	LOSS	0.445
IM95m	E452K	WT	*0.510*
AGS	E453K	WT	0.552
NCI-N87	WT	WT	*1.037*
23132/87	WT	WT	*1.671*
MKN1	E545K	WT	2.421
SNU-620	WT	WT	*3.384*
SNU-638	WT	WT	*4.523*
SNU-1	WT	WT	*5.258*
SNU-601	WT	WT	*5.938*
SNU-668	WT	WT	*6.003*
HS746T	WT	WT	*6.084*
KATO III	WT	WT	7.267
SNU-484	WT	WT	*7.392*
SNU-16	WT	WT	*11.097*
OCUM-1	WT	WT	*14.515*
NUGC-3	WT	WT	*21.873*
AZ521	WT	WT	*25.448*
SNU-216	WT	WT	*30.000*
NUGC-4	WT	WT	*30.000*
SNU-5	WT	WT	*30.000*
GTL-16	WT	WT	*30.000*
MKN74	WT	WT	*30.000*
PAMC82	WT	WT	*30.000*

A panel of GC cell lines with differing genetic backgrounds were screened in a standard MTS cell proliferation assay. The PI3KCA mutation and PTEN expression status were determined by ASPCR and IHC respectively.

### Prevalence of PI3KCA mutations and PTEN loss in Chinese GC

**Figure 1:**
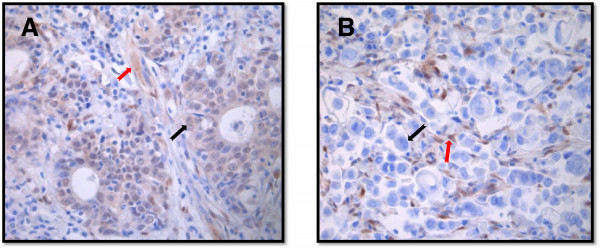
**Representative images from PTEN IHC staining. (A)**. Positive PTEN staining was observed in the cytoplasm and nuclei of the tumor cells (black arrow) and stroma cells (red arrow). **(B)**. Negative PTEN staining in the tumor cells (black arrow) and positive staining in the stroma cells.

Next we sought to identify GC patients with both PI3KCA mutations and PTEN loss, who will benefit the most from AZD5363 therapy. The hotspot mutations of PI3K, Braf and Kra*s* genes and loss of PTEN was screened in a cohort of 150 Chinese GC samples by using mutation specific PCR and IHC respectively. As shown in Table 2, PI3KCA mutations were identified in the samples from 4 patients (3 with grade 3 and 1 with grade 2 diseases), representing a mutation rate of 2.7% (4/150). Besides PI3K activation through mutation, loss of PTEN represents another mechanism through which the PI3K/AKT pathway can become activated. Thus, we also investigated the expression status of PTEN in GC. A total of 61 qualified tumor samples from the same cohort of Chinese GC were examined by IHC staining using an anti-PTEN antibody. As shown in Table 2 and Figure 1, the loss of PTEN protein expression was found in 23% (14/61) of the tested samples, consistent with the reported rate of 20% [20]. Further sequencing analysis of the 61 samples indicated that PTEN loss overlapped with Braf mutation in one case, but was mutually exclusive with PI3KCA and Kras mutations (Table 3).

**Table 2:** PI3KCA mutations and PTEN loss in Chinese GC

**Mutation**	**Assay**	**Status (%)**
PI3KCA (hot spots)	ASPCR	2.7% (4/150)
PTEN loss	IHC	23% (14/61)

**Table 3:** Genetic background of GC with PTEN loss

**Sample ID**	**PTEN IHC staining results**	**PIK3CA mutation status**	**BRAF mutation status**	**KRAS mutation status**
802204	(-)	Wild type	Wild type	Wild type
1007947	(-)	Wild type	V600E	Wild type
44256	(-)	Wild type	Wild type	Wild type
34711	(-)	Wild type	Wild type	n/a
608902	(-)	Wild type	Wild type	Wild type
60077	(-)	Wild type	Wild type	Wild type
606186	(-)	Wild type	Wild type	n/a
803776	(-)	Wild type	Wild type	Wild type
1007953	(-)	Wild type	Wild type	Wild type
708053	(-)	Wild type	Wild type	Wild type
708025	(-)	Wild type	Wild type	n/a
16627	(-)	Wild type	Wild type	n/a
505496	(-)	Wild type	Wild type	n/a
508745	(-)	Wild type	Wild type	Wild type
600460	(+)	E542K/E545D*	Wild type	n/a
802664	(+)	Wild type	Wild type	G12D*

*The PI3KCA and KRAS mutations were mutually exclusive from PTEN loss.

### Anti-tumor efficacy of AZD5363 in gastric PDGCX mouse models with PI3KCA mutation or PTEN loss

**Figure 2:**
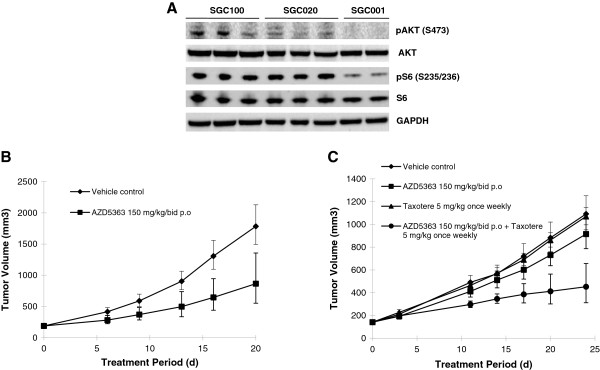
**AZD5363 treatment results in significant inhibition in PDGCX mouse models. (A)**. Activation of AKT signaling pathway in SGC100 and SGC020 PDGCX models. The basal levels of phosphor AKT (S473) and phosphor S6 (S235/236) in PDGCX tumors were measured by Western blot analysis. **(B)**. Anti-tumor efficacy of AZD5363 monotherapy in SGC100 PDGCX model. AZD5363 was administered by oral gavage twice (bid) daily to nu/nu mice bearing established PDGCX SGC100 model with PI3KCA mutant tumors at 150 mg/kg single agent. Tumor volumes were monitored and plotted against time. **(C)** Anti-tumor efficacy of AZD5363 in combination with Taxotere in PDGCX SGC020 model. AZD5363 was administered by oral gavage twice (bid) daily to nu/nu mice bearing established PDGCX SGC020 model with PTEN null tumors at 150 mg/kg in combination with Taxotere at 5 mg/kg weekly. Tumor volumes were monitored and plotted against time.

The lack of GC patients with both PI3KCA mutations and PTEN loss and the high prevalence of PTEN loss observed in GC triggered us to investigate the response of GC with PTEN loss to AZD5363. However, due to the lack of GC cell lines with PTEN loss and wild-type PI3K, we screened 15 gastric PDGCX mouse models established from surgical samples of Chinese GC patients. The expression levels of PTEN protein were measured by IHC staining and genomic PTEN aberrations were detected by MLPA analysis respectively. PI3KCA hotspot mutations were screened by direct sequencing. As indicated in Table 4, SGC020, a PDGCX model with a PTEN exon 2-6 gene deletion and undetectable PTEN protein expression and SGC100, a PDGCX model harboring a PI3KCA H1047R activating mutation and positive PTEN staining, were both identified for AZD5363 anti-tumor efficacy study. As shown in Figure 2A, higher levels of basal phosphor AKT and phosphor S6 were detected by Western blot in SGC100 and SGC020 tumors compared to that in the SGC001 PDGCX tumors with PI3K and PTEN wild-type status, indicating the up-regulation of AKT signal pathway in SGC100 and SGC020 models.

**Table 4:** Characterization of PDGCX mouse models

**Model ID**	**Her2 amp**	**PTEN analysis**		**PI3KCA mutation**
	**aCGH**	**MLPA**	**IHC**	**Sequencing**
SGC020	(-)	Exon2-6 deletion	(-)	Wild-type
SGC100	(-)	N/A	70% ++ (C&N)	H1047R

Next we tested the response of SGC100 and SGC020 models to AZD5363. As shown in Figure 2B and 2C, AZD5363 single agent treatment resulted in 60% tumor growth inhibition in SGC100 model (P < 0.0001) but had only marginal effects (23% tumor growth inhibition, p < 0.002) in the PTEN null SGC020 model. AZD5363 treatment in this study was well tolerated and did not result in significant body weight loss. These data indicate that PI3KCA mutations, but not PTEN loss, predicate the sensitivity to AZD5363 in GC.

Chemotherapy is the current standard of care for GC. In additional effort, we preformed *in vitro* combination of AZD5363 with the commonly used chemotherapy agents in GC including Taxotere, SN-38 and Oxaliplatin in a number of GC cell lines with both PI3KCA mutation and PTEN loss (HGC27), PI3KCA mutation alone, and PI3K and PTEN wild-type status. Our data showed that the combination of AZD5363 with Taxotere, SN-38 and Oxaliplatin resulted in additive or slightly synergistic effect regardless of the mutation status in PI3K gene (Additional file 1: Table S1).

Previous reports have suggested a role for PTEN loss in chemotherapy resistance [15]. Therefore, we next tested whether PTEN loss contributed to Taxotere resistance, one of the major chemotherapy agents used clinically in GC [21]. As shown in Figure 2B, Taxotere at a human equivalent dose of 5 mg/kg weekly had no effect on tumor growth in the SGC020 model with PTEN loss. In contrast, combinations of AZD5363 and Taxotere resulted in significant tumor inhibition (67%, p < 0.05) in the PDGCX model, supporting a potential combination strategy for the treatment of GC with PTEN loss. In addition, the induction of caspase3/7 by combination of AZD5363 with Taxotere, the hallmark of cell apoptosis, was observed in multiple tested cell lines, suggesting the anti-tumor effect of AZD5363 with Taxotere by induction of apoptotic cell death (Additional file 1: Table S1).

### Pharmacodynamic modulation of AKT signaling by AZD5363 correlates with anti-tumor activity

**Figure 3:**
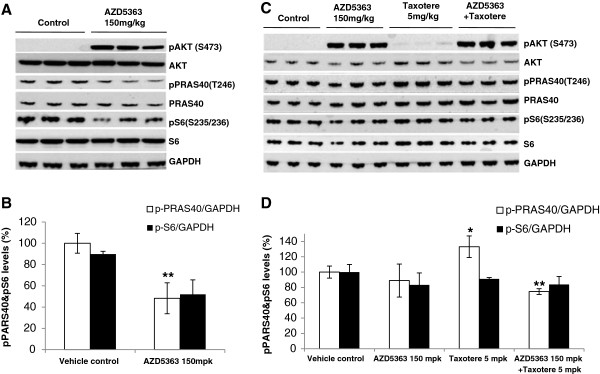
**Pharmacodynamic study of tumors treated with AZD5363 or in combination with Taxotere. (A)** and **(B)**. Suppression of AKT downstream signalling by AZD5363 in SGC100 PDGCX tumors with PI3KCA muttion. The tumor bearing mice were treated with AZD5363 (150 mg/kg, bid) for 25 days. Tumors samples were collected two hours post-final dose and were subjected to western blot analysis for phospho-AKT (S473), phospho-PRAS40 (T246) and phosphor S6 **(A)**. The expression levels were quantified by gel imaging scan and expressed as mean + SD **(B)**. **(C)** and **(D)**. Mudulation of AKT downstream signalling by AZD5363 and Taxotere in SGC020 PDGCX tumors with PTEN loss. The tumor bearing mice were treated with AZD5363 (150 mg/kg, bid) in combination with Taxotere (5 mg/kg weekly) for 25 days. Tumors samples were collected two hours post-final dose and were subjected to western blot for phospho-AKT (S473), phospho-PRAS40 (T246) and phosphor S6 **(C)**. The expression levels were quantified by gel imaging scan and expressed as mean + SD **(D)**. Students’ t-tests were used to compare modulation in the treatment group with the control group. Statistical tests were two sided, with P < 0.05 considered significant (*).

To understand the mechanism of AZD5363 anti-tumor efficacy and its combination with Taxotere in SGC020 and SGC100 models, tumor samples were collected two hours post-last dose of AZD5363. Tissues lysates were subjected to Western blot analysis of PRAS40 and S6 phosphorylation, both downstream targets of AKT signaling. As shown in Figure 3, AZD5363 single agent treatment led to up-regulated pAKT in both SGC100 and SGC020 PDGCX models, indicating the engagement of AZD5363 with its specific target [17]. It is noteworthy that the undetectable pAKT (S473) in the untreated SGC100 and SGC020 tumors was due to a shorter western blot exposure since very strong signals were detected in the AZD5363 treated samples. Interestingly, the suppression of AKT downstream signaling monitored by pPRAS40 (52%) and pS6 (42%) was only observed in the PI3KCA mutant PDGCX model (SGC100), but not in the PTEN null PDGCX model (SGC020), correlating with AZD5363 anti-tumor efficacy. Consistent with our recent observations in cell cultures (data not shown), Taxotere treatment led to a moderate increase of pPRAS40 (Figure 3C/3D) in SGC20 model and the addition of AZD5363 blocked this induction. These results further support the activation of AKT signaling as a resistance mechanism to chemotherapy agents, and strongly suggest AKT inhibition as a viable sensitization approach.

## Discussion

Emerging evidence supports a critical role for the activation of the PI3K/AKT signaling network in both tumorigenesis and drug resistance. A number of novel small molecule inhibitors targeting PI3K, AKT and mTOR are at different stages of drug development for the treatment of a variety of solid tumors, including GC [22]. Everolimus, an mTOR inhibitor approved for breast cancer, neuroendocrine tumors of pancreatic origin and subependymal giant cell astrocytoma, has been tested in GC patients in a phase II and phase III trial. In the recent phase II trial, everolimus demonstrated anti-tumor activity with a response rate of 3.7% (2/44) and disease control rate (DCR) of 38.9% (17/44) [23]. Although the trial failed to achieve its primary objective (30% of 4-month progression-free survival (PFS) rate), the observed association between high expression of pS6 (Ser240/4) at baseline and higher DCR and prolonged PFS warrant further trials with a molecular stratification-based patient selection strategy.

Recently we reported the development of AZD5363, a potent pan-AKT kinase inhibitor, which is currently undergoing phase I clinical investigation. Our previous results demonstrated that cancer cells harboring PI3KCA activating mutations, PTEN mutations (loss or mutations), or Her2 amplification were highly sensitive to AZD5363 [17]. This sensitivity was further confirmed in several xenograft tumor models. Interestingly, we found that HGC27, a GC cell line with PI3KCA mutation and PTEN loss was the most sensitive line amongst all those tested, both *in vitro* and *in vivo*. Because PTEN loss is more prevalent than PI3KCA mutations and the frequency of these concurrent aberrations is very low, it is intriguing to understand whether the loss of function of PTEN alone is sufficient to drive the response to AKT inhibition.

PTEN phosphatase is a novel tumor suppressor and negatively regulates the PI3K/Akt signaling network [24]. PTEN functional loss can be attributed to inactivating gene mutations, chromosomal deletions and promoter methylation, all of which are implicated in multiple cancer types including GC [25]. In comparison to PI3KCA mutations, the lack of detectable PTEN protein expression by IHC staining was more frequently observed in GC with reported rates of between 20-36% [11,13]. However, the role of PTEN in driving cancer development and the response to agents targeting the PI3K/AKT pathway is yet to be fully understood. In this report, we screened 15 primary xenograft models derived from primary GC tumors and identified one model with PI3KCA activating mutation and another with undetectable PTEN expression. When treated with AZD5363, potent anti-tumor responses were observed in the PI3KCA mutant PDGCX model, consistent with the cell panel data. In contrast, no significant efficacy was observed in the PDGCX model with PTEN loss, suggesting a minor role for PTEN loss in driving tumorigenesis in GC. However, when a PTEN null PDGCX model was treated with a combination of AZD5363 and taxotere, a synergistic effect was achieved compared to treatment with either single agent. This is consistent with previous observations by us and others [17,26]. In the Her2 positive BT474 model, a combination of AZD5363 with Taxotere led to complete tumor regression. Similarly, Lin et al showed that administration of another AKT kinase inhibitor, GDC-0068, in combination with Taxotere induced tumor regression in the PC-3 prostate xenograft model with a homozygous deletion of PTEN, at doses where each single agent only caused modest tumor growth delay. To further understand the underlying molecular mechanism for the combination effect in the PTEN null PDGCX model (SGC020), pAKT modulation and downstream signaling was studied. Consistent with previous observations, increased AKT phosphorylation was observed following AZD5363 treatment, suggestive of target engagement. However, we did not observe anti-tumor efficacy using AZD5363 monotherapy, nor modulation of pPRAS40 and S6. The reason for the inability of single agent AZD5363 to elicit efficacy in a PDGCX model (SGC020) is not currently fully understood. As the tumor samples used for the pharmacodynamic study were collected after a multiple dosing regimen, we postulate that there may be dynamic subtleties within the AKT signaling pathway and that further study using a single dose of AZD5363 over a dynamic time course may provide additional insight. Consistent with our own results from cell culture studies (data not shown), the expression levels of pPRS40 and pS6 were moderately upregulated by Taxotere in a PDGCX SGC020 model and treatment with AZD5363 blocked this activation, suggesting a possible mechanism for the anti-tumor efficacy observed in the combination study.

PI3KCA hotspot mutations in GC have been previously reported by several groups. Mutation frequencies range from 10.6%% to 15.9% [27,28] in Caucasian and 4.3% to 7.1% in Asian GC [12,29]. This apparent discrepancy between Western and Eastern populations may be due to geographical differences, as shown for the situation with EGFR mutation in lung cancer [30]. In a separate study we found that the mutations in a number of oncogenes, including PI3KCA mutations, are enriched in advanced stage and genomically unstable patients (manuscript in preparation). The low frequency (2.7%, 4/127) of PI3KCA mutation detected in our study may be due to the relatively small sample size related to disease stage and genomic instability status.

The observations described in this study were supported by emerging data from our ongoing two AZD5363 phase I clinical trials [31]. As a monotherapy, AZD5363 was generally well tolerated when administrated using intermittent doses of 480 mg twice daily, with four days on and three days off. The pharmacokinetic studies indicated that exposures achieved in patients were comparable to those achieved at efficacious doses used in our preclinical animal studies. Reductions in pPRAS40 and pGSK3β in plucked hair and blood samples were observed in 30% of patients. To date, partial responses have been observed in two treated patients, harboring tumor mutations in either AKT1 or PI3KCA. Given the high prevalence of PTEN loss in gastric cancer, the synergistic combination effect of AZD5363 with Taxotere in the PTEN-loss primary model warrants further clinical trial for potential application of AKT inhibitors for the treatment of patients with PTEN null tumors.

In conclusion, AZD5363, a potent and selective small molecule AKT inhibitor, demonstrates the effectiveness to suppress growth of PI3KCA mutant GC cells *in vitro* and PDGCX model *in vivo*. It reverses the *de novo* resistance to Taxotere in a PTEN loss PDGCX model. These results point out a potential new strategy for treatment of subsets of GC patients with AKT inhibitors.

## Competing interests

The authors declare that they have no competing interests.

## Authors’ contributions

DY participated in study design and coordination and drafted the manuscript. JL, MZ, JZ performed the establishment of animal tumor models and efficacy study. SH, YX, YB, LT and HW completed the PD analysis and in vitro cell proliferation and combination studies. YL and XY completed immunochemistry staining. BD and QJ conceived of the study and participated in its design. All authors read and approved the final manuscript.

## Supplementary Material

Additional file 1: Table S1 *In vitro* combination of AZD5363 with chemotherapy agents in GC cells. Click here for file
