# Supplementary material for: The AKT inhibitor AZD5363 is selectively active in PI3KCA mutant gastric cancer, and sensitizes a patient-derived gastric cancer xenograft model with PTEN loss to Taxotere
Source: J Transl Med. 2013 Oct 2;11:241. doi: 10.1186/1479-5876-11-241 (PMC3850695; doi:10.1186/1479-5876-11-241)
Supplement: Additional file 1: Table S1 — In vitro combination of AZD5363 with chemotherapy agents in GC cells. [file 1479-5876-11-241-S1.docx]

**Supplementary table 1. *In vitro* combination of AZD5363 with chemotherapy agents in GC cells**

|  | IC_50_ (μmol/L) | | | | Genotypes | | | Combination index | | | Caspase-3/7 |
| --- | --- | --- | --- | --- | --- | --- | --- | --- | --- | --- | --- |
| Cell line | AZD5363 | Oxalipatin | SN-38 | Taxotere | PI3K | PTEN | Ras | AZD5363+Taxotere | AZD5363+SN-38 | AZD5363+Oxalipatin | Maximum fold induction at tested concentration |
| HGC27 | 0.4452 | 1.3440 | 0.0182 | 0.0010 | E453K | HM | WT | 1.292 | 1.050 | 0.856 | 6.6 |
| MKN1 | 2.4210 | 2.0190 | 0.0274 | 0.0014 | E545K | WT | WT | 0.876 | 0.561 | 0.721 | 2.3 |
| IM95m | 0.5097 | 0.8257 | 0.0457 | 0.0026 | E542K | WT | WT | 0.871 | 0.811 | 1.362 |  |
| AGS | 0.5523 | 0.4442 | 0.0107 | 0.0013 | E453K | WT | K-Ras G12D | 0.651 | 0.942 | 1.248 |  |
| MKN74 | 30.0000 | 19.4300 | 0.1992 | 0.0051 | WT | WT | WT | 0.72 | 0.389 | Can't calculate | 1.4 |
| NUGC4 | 30.0000 | 0.6957 | 0.0199 | 0.0019 | WT | WT | WT | 0.702 | 0.604 | 1.103 | 3.6 |

**Supplementary method**

Cells were seeded at a density of 1 to 4 Χ 10^3^ per well in 96-well plates overnight. The drugs at different concentrations, either as a single agent or in combination, were added to the wells as concurrent treatment. 72h after compound treatment, cells were stained with Sytox green(Invitrogen) for 2h and read at Acumen X3 for dead cell number. The cells were further treated with 0.25% saponin (made up in Tris EDTA 5mM, pH7.0, sigma) for overnight, then the total cell number was measured by a Acumen X3. Live cell number was determined by deduction of the dead cell number from the total cell number. The nature of the drug interaction was evaluated by using the combination index (CI) according to the method of Chou and Talalay (Adv Enzyme Regul. 1984; 22:27-55).
